# Risk Factors and Regression of Coronary Aneurysms in Infants With Kawasaki Disease

**DOI:** 10.1155/cdr/9988778

**Published:** 2025-11-11

**Authors:** Yunxia Liu, Yumao Zhang, Ying Xie, Weibin Li, Hong Wei, Lin Liu, Zhenheng Ou, Boning Li, Meng Li

**Affiliations:** ^1^Department of Cardiology, Shenzhen Children's Hospital, Shenzhen, China; ^2^Department of Pharmacy, The Eighth Affiliated Hospital of Sun Yat-sen University, Shenzhen, China; ^3^The Office of Drug Clinical Trial Institutions, Shenzhen Children's Hospital, Shenzhen, China; ^4^Department of Pharmacy, Shenzhen Children's Hospital, Shenzhen, China

**Keywords:** coronary aneurysm, infants, Kawasaki disease, regression, risk factors

## Abstract

**Purpose:**

The purpose of this study is to analyze clinical characteristics of patients with Kawasaki disease (KD) aged below 1 year and identify risk factors for coronary aneurysm (CA) and its associated prognosis.

**Methods:**

A retrospective study enrolling infants (under 1 year of age) with KD admitted to a children's hospital between January 1, 2012, and August 30, 2024, was conducted. Patients were divided into two groups based on CA presence. Clinical records, including demographics, clinical manifestations, treatments, laboratory parameters, and cardiac ultrasound examination, were collected and analyzed.

**Results:**

Of 381 infants with KD, 96 developed CA. Male sex (adjusted odds ratio [aOR] = 1.982, *p* = 0.04), duration of fever (aOR = 1.143, *p* = 0.03), illness days of initial intravenous immunoglobulin (IVIG) (aOR = 1.319, *p* < 0.01), and higher C-reactive protein (CRP) (aOR = 1.007, *p* = 0.02) were associated with CA development in infants with KD in multivariable analysis. Specifically, an increase of 10 mg/L in CRP is associated with a 7.2% elevation in risk of CA development. Among patients diagnosed with CA, 30, 42, and 24 had small aneurysm (sAN), medium aneurysm (mAN), and giant aneurysm (gAN), respectively. The complete regression proportion and regression times of sAN, mAN, and gAN were 86.7%, 76.2%, and 33.3% and 3.9 ± 9.2, 12.8 ± 17.2, and 20.7 ± 11.2 months, respectively.

**Conclusions:**

CA incidence was higher in infants with KD and was associated with male sex, fever duration, days of initial IVIG, and higher CRP. The ability and time of regression depend on the size of the CA; however, giant CA in infant patients has a greater tendency to regress than those in older patients with KD.

## 1. Introduction

Kawasaki disease (KD), also known as mucocutaneous lymph node syndrome, is an acute febrile illness that predominantly affects children under 5 years of age [1]. KD primarily induces systemic vasculitis of small- to medium-sized vessels, which is associated with a risk of developing coronary aneurysms (CAs), which are prone to coronary thrombus formation due to coronary arterial endothelial injury, acute hypercoagulability, and blood stasis [2, 3]. Consequently, coronary thrombosis can lead to acute myocardial infarction or heart failure, and KD has recently become a major cause of acquired heart disease in children and ischemic cardiomyopathy in adults. Furthermore, epidemiological investigations have indicated that the incidence of KD has gradually increased in recent years, as awareness of KD has improved [4, 5]. Among patients with KD, the highest incidence rate of KD was reported in infants under 1 year of age [5, 6], accounting for 28.3% of all patients with KD in one Italian cohort [7]. These patients have a higher prevalence of cardiac complications, including CA, due to their atypical clinical symptoms and delayed diagnosis [5, 6].

Delayed diagnosis and incomplete clinical manifestations have been suggested as major contributors to CA development, possibly due to an association with delayed intravenous immunoglobulin (IVIG) treatment, which has been demonstrated to decrease the risk of CA development [2]. Thus, the early diagnosis of KD and verification of risk factors for CA development in 1-year-old patients could have important clinical implications. In this context, the present study is aimed at analyzing risk factors for CA development in patients with KD aged <1 year. Furthermore, knowledge regarding the clinical course of CA regression in these populations is limited, which is a matter of concern for clinical practice and patients' families, as it determines the medication choices and treatment course, particularly the use of antiplatelet medicine. Therefore, the present study is also aimed at exploring the prognosis of CA in patients under 1 year of age by analyzing echocardiographic follow-up results.

## 2. Methods

### 2.1. Study Population

This retrospective study was conducted at Shenzhen Children's Hospital through the collection and analysis of the clinical records of infant patients (defined as under 1 year old) who were diagnosed with KD with or without CA between January 1, 2012, and August 30, 2024. The exclusion criteria included age>1 year, incomplete laboratory and echocardiography results, and diagnosis at other hospitals without precise treatment records. Patients with KD were divided into two groups: The control group comprised those without CA (non-CA group), and the case group comprised those with CA (CA group). This study was approved by the Institutional Research and Ethics Committee (No. 202404902). Informed consent was waived by the institutional review board.

### 2.2. Definition and Classification

The diagnosis of KD was made according to the criteria of the American Heart Association (AHA) [8], based on the following factors: (1) persistent fever lasting more than 5 days, (2) bilateral conjunctival congestion, (3) asymmetric lymph node enlargement, (4) changes in the lips and oral mucosa, (5) polymorphic rash, and (6) acute swelling of the fingertips and distal toes, with peeling along the fingertips and distal toes during the recovery period. Complete or typical KD can be diagnosed in patients who have a fever for 5 days and meet more than four of the major criteria. Incomplete or atypical KD is often diagnosed in patients who lack the full clinical features of typical KD, and the diagnosis of incomplete KD is thus based on the 2017 criteria of the AHA. IVIG-resistant KD was defined as recrudescent or persistent fever for at least 36 h after the completion of the first IVIG infusion [8].

The definition of CA was based on the absolute value of the inner blood vessel diameter and/or *z*-score according to the criteria of the AHA [8]. With the help of a model based on data from a large cohort of Chinese healthy children, the maximum internal diameter of each artery in the coronary arteries was converted to a *z*-score corrected by the body surface area [9]. In brief, small aneurysm (sAN) was defined by a *z*-score of ≥+2.5 to <+5 or an absolute dimension of ≥3 to <4 mm, medium aneurysm (mAN) as a *z*-score of ≥+5 to <+10 or an absolute dimension of ≥4 to <8 mm, and giant aneurysm (gAN) as a *z*-score of ≥+10 or an absolute dimension of ≥8 mm. In this study, peripheral artery dilation or aneurysm in the arterial system other than the coronary circulation was defined by an increase in vessel diameter exceeding 50% of the normal reference value, which is diagnostically classified as an aneurysm. Conversely, if the diameter expansion is less than 50%, the condition is referred to as arterial dilation.

Echocardiograms were performed according to a standard protocol applied to all patients with KD. Echocardiographic evaluation is typically conducted at the time of initial diagnosis and every 2–3 days during the acute phase (within 2 weeks of onset). For high-risk patients, such as those unresponsive to IVIG or those with CA, echocardiographic assessments are performed more frequently and at similar intervals. Following discharge, routine follow-up examinations are generally scheduled at intervals of 1 week and at 1, 3, 6, and 12 months and even longer postdischarge. For patients with CA, the frequency of follow-up visits is intensified to ensure closer monitoring and timely intervention.

Complete CA regression was defined as the return of the absolute diameter or *z*-score of the coronary artery to the normal range (absolute diameter <3 mm or *z*-score <2.5). Partial regression of CA was defined as recovery from gAN to mAN or sAN or recovery from mAN to sAN. Unchanged CA was defined as the maintenance of the classification.

### 2.3. Data Collection

Clinical manifestations, age, sex, weight, diagnosis time (Illness Day 1 means the first day of fever), response to IVIG therapy, laboratory parameters prior to initial IVIG infusion (white blood cells, [WBCs], neutrophil [NEU], lymphocyte [LYM], hemoglobin [HB], platelet [PLT], C-reactive protein [CRP], albumin [ALB], total protein [TP], Na+, lactic dehydrogenase [LDH], erythrocyte sedimentation rate [ESR], brain natriuretic peptide [BNP], ferritin [FER], procalcitonin [PCT], alanine aminotransferase [ALT], aspartate aminotransferase [AST], and fibrinogen degradation products [FDPs]), and coronary artery status during acute phase and recovery phase (including affected coronary branches such as the left main [LM] coronary artery, proximal and distal left anterior descending artery [LAD], left circumflex [LCX], and proximal, middle, and distal right coronary artery [RCA], the time of presence and regression of CA, and inner diameter peak value) were recorded and compared between the non-CA and CA groups. The follow-up echocardiographic findings in the CA group were analyzed.

### 2.4. Treatment Protocol

Following the AHA guidelines, initial treatment with IVIG (2 g/kg) as a single infusion and aspirin (30–50 mg/kg/day in the acute phase and 3–5 mg/kg/day 2–3 days after the patients were afebrile) was administered to patients with a KD diagnosis [8]. Combined antiplatelet and anticoagulation therapies were administered based on patient risk stratification. If patients were IVIG-resistant or unresponsive, one or more IVIG infusions (2 g/kg) alone or combined with methylprednisolone (MP) (2 mg/kg/day) were administered at the discretion of the physician.

### 2.5. Statistical Analysis

Means ± standard deviations (SDs) are shown for continuous variables and proportions for categorical variables. Student's *t*-test and the chi-square test were used for continuous and categorical variables, respectively, as appropriate. Continuous variables were presented as means ± standard deviations, while categorical variables were summarized as counts and percentages. Comparative analyses between groups were performed using Student's *t*-test for continuous variables and either chi-square or Fisher's exact test for categorical variables, depending on appropriateness. Candidate predictors with a *p* value < 0.20 in univariate analysis were retained, and essential clinical variables identified from prior literature were incorporated into an unconditional logistic regression model to ensure a balance between statistical robustness and clinical relevance. Missing data were addressed through multiple imputation using chained equations, incorporating sex, age, and weight as predictors. The primary logistic regression analysis was then performed on the pooled imputed datasets. Sensitivity analysis was performed by refitting the logistic regression model after listwise deletion of observations with missing values. Prior to model fitting, multicollinearity among predictors was assessed using variance inflation factors (VIFs), with variables exhibiting VIF ≥ 5 excluded from the analysis. Model performance was assessed in terms of discrimination and calibration. Discrimination was quantified using Nagelkerke's pseudo-*R*^2^, while calibration was evaluated via the Hosmer–Lemeshow goodness-of-fit test. Adjusted odds ratios (aORs) with 95% confidence intervals were reported for all covariates in the final model. Laboratory indicators were generally treated as continuous variables within the model, except for those with established clinical thresholds, such as hypoproteinemia and anemia, which were dichotomized. For continuous variables, optimal cutoff points were determined based on the coordinates closest to the top-left corner of the receiver operating characteristic (ROC) curve. The Kaplan–Meier curves were plotted to illustrate the regression of the three CA subtypes during follow-up, with intergroup differences assessed by the log-rank test. All statistical analyses were performed using SPSS Statistics for Windows, Version 28.0 (IBM Corp., Armonk, New York). Statistical significance was set at a two-sided *p* value < 0.05.

## 3. Results

### 3.1. Demographic Data

**Figure 1:**
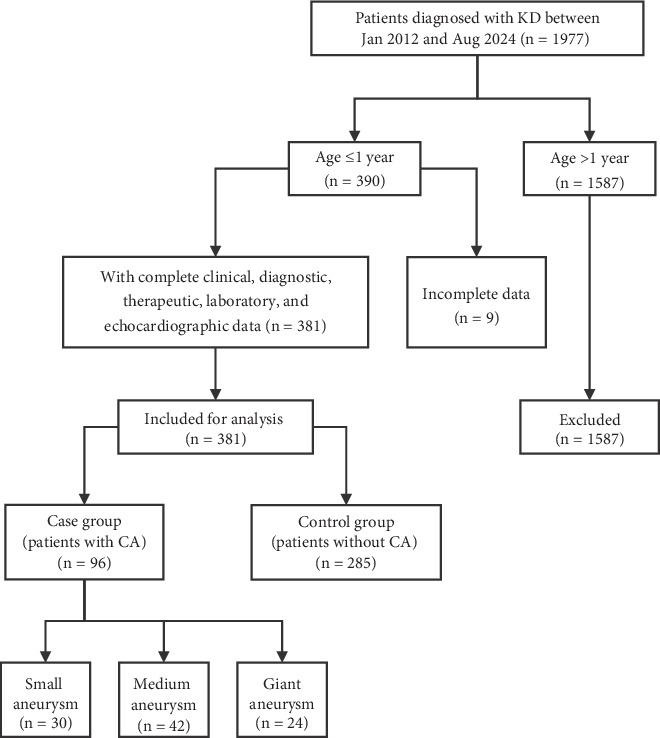
Patient screening flow diagram.

During the study period, 390 patients under 1 year of age were diagnosed with KD, of whom nine patients were excluded due to incomplete information. Finally, 285 patients with KD and without CA (non-CA group) and 96 patients with KD and CA (CA group) were included (Figure 1). Among these patients, 22 patients in the non-CA group and 35 patients in the CA group were referred from external medical centers, while 19 patients were diagnosed with CA at the time of admission.  Table 1 presents the demographic data of patients in the non-CA and CA groups.

The study included 187 (65.6%) male patients (male-to-female ratio 1.9:1) in the non-CA group and 69 (71.9%) male patients (male-to-female ratio 2.6:1) in the CA group. The fever durations in the two groups were 7.1 ± 2.7 and 9.9 ± 3.4 days, respectively, showing a significant difference (*p* < 0.05). The respective initial days of IVIG treatment postfever onset were 6.2 ± 2.0 and 8.8 ± 4.1 days, showing a significant difference (*p* < 0.05). Furthermore, initial IVIG treatment within 10 days of KD onset was received in 266 (93.3%) and 59 (61.5%) patients in the non-CA and CA groups, respectively (*p* < 0.05). The proportion of incomplete and IVIG-resistant KD cases was significantly higher in the CA group than in the non-CA group (*p* < 0.05). All patients presented with a fever. The frequencies of clinical signs, including conjunctival congestion, changes in the lips and oral mucosa, and polymorphic rash, were significantly different between the two groups (*p* < 0.05). No patients in the non-CA group showed peripheral vascular dilation or aneurysms, while nine patients with CA had peripheral vascular dilation or aneurysms, as detailed in Table 1.

### 3.2. Treatments

In the non-CA group, all patients (*n* = 285, 100%) received the first dose of IVIG, with 27 patients (9.5%) concurrently receiving corticosteroid therapy. Similarly, in the CA group, all patients (*n* = 96, 100%) received the first dose of IVIG, with five patients (5.2%) simultaneously undergoing corticosteroid treatment (Table 2). Among the 58 IVIG-resistant patients in the non-CA group, 48 (82.8%) received a second dose of IVIG. Of these, 23 (47.9%) also received corticosteroid therapy, while five patients received three IVIG doses. The remaining 10 patients (17.2%) were treated exclusively with corticosteroids. In the CA group, there were 36 patients who were IVIG-resistant; 31 patients (86.1%) received a second dose of IVIG, with 22 (71.0%) of them concomitant with corticosteroid therapy; and 10 patients underwent three doses of IVIG. The remaining five patients (13.9%) were treated with corticosteroids only (Table 2). In addition to the IVIG and steroids, there were five patients who used infliximab due to the continuous coronary dilation. This analysis highlights the heterogeneity in treatment strategies for KD, particularly in cases unresponsive to IVIG, underscoring the need for a unified therapeutic protocol to optimize patient outcomes.

For antiplatelet treatments, 125 patients received single antiplatelet therapy with aspirin, dipyridamole, or clopidogrel, and 160 patients received dual antiplatelet therapy (aspirin and dipyridamole) in the non-CA group (Table 2). In the CA group, in patients with sAN, 11 patients received single antiplatelet therapy with aspirin or dipyridamole, and 21 patients received dual antiplatelet therapy with aspirin and dipyridamole. In patients with mAN, four of them received single antiplatelet therapy with aspirin or dipyridamole, 32 received dual antiplatelet therapy, and four received dual antiplatelet and warfarin. In patients with gAN, eight of them received dual antiplatelet therapy, 10 received aspirin and warfarin, and six received dual antiplatelet therapy with warfarin (Table 2).

### 3.3. Laboratory Variables

The laboratory results reflect the degree of inflammation in patients with KD; therefore, the laboratory peak values of patients with KD with or without CA during the acute phase were evaluated. The PLT, CRP, and ESR were significantly higher in the CA group than in the non-CA group, whereas the ALB level in the CA group was significantly lower than that in the non-CA group (Table 3). In addition, WBC count and HB levels tended to differ between the two groups (*p* = 0.06, Table 3).

### 3.4. Echocardiographic Evaluation in Patients With KD and CA

Among patients with CA (*n* = 96), 30 (31.2%), 42 (43.8%), and 24 (25.0%) patients had sAN, mAN, and gAN, respectively. The median time for initial coronary dilation (*z* > 2), CA formation, and maximum dilation in all patients with KD and CA was 10 days (4–44 days), 13 days (4–44 days), and 16 days (4–432 days), respectively. The median time for initial coronary dilation in the sAN, mAN, and gAN groups was observed to be 9 days (4–26 days), 10 days (4–44 days), and 13 days (4–26 days), respectively. The median time for CA formation in the sAN, mAN, and gAN groups was observed to be 9 days (4–26 days), 10 days (5–44 days), and 14.5 days (4–30 days), respectively. The median time for maximum coronary dilation in the sAN, mAN, and gAN groups was observed to be 9.5 days (4–30 days), 14 days (5–47 days), and 25 days (4–432 days), respectively (Table 4).

**Figure 2:**
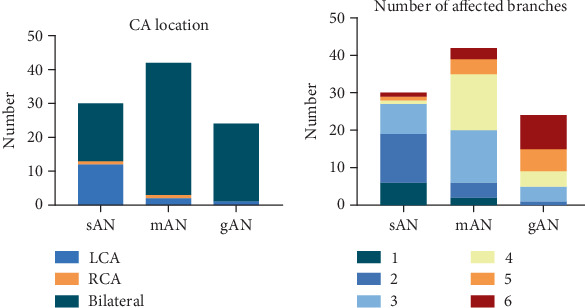
The location of coronary aneurysm (CA) and the number of affected branches in three subgroups. (a) The distribution of CA among patients with sAN, mAN, and gAN. (b) The number of affected branches in patients with sAN, mAN, and gAN. CA, coronary aneurysm; sAN, small aneurysms; mAN, medium aneurysms; gAN, giant aneurysms.

In most patients (*n* = 79, 82.3%), CA was associated with bilateral coronary lesions, which were observed in 17 (56.7%), 39 (92.9%), and 23 (95.8%) patients with sAN, mAN, and gAN, respectively (Figure 2a). Fewer than three branches were affected in 90.0% of patients in the sAN group, three and four branches were affected in 69.0% of patients in the mAN group, and more than three branches were affected in 95.8% of patients in the gAN group (Figure 2b).

### 3.5. Risk Factors Associated With CA Development

Univariate analysis identified fever duration, illness days at initial IVIG treatment, IVIG resistance, complete KD, WBC, NEU, LYM, platelet count, CRP level, ALB < 30 g/L, HB < 95 g/L, TP, and AST/ALT ratio (*p* < 0.2) as potential risk factors associated with CA (Table S1). We subsequently conducted further unconditional logistic regression analyses by including these potential factors and sex, weight, age, and PCT level in the model (Table 5). Finally, male sex (aOR = 1.982, *p* = 0.04), duration of fever (aOR = 1.143, *p* = 0.03), illness days at initial IVIG treatment (aOR = 1.319, *p* < 0.01), and higher CRP (aOR = 1.007, *p* = 0.02) were identified as risk factors for CA in patients with KD. Specifically, an increase of 10 mg/L in CRP is associated with a 7.2% elevation in risk of CA development. The logistic regression model was constructed using the imputed dataset, with the Hosmer–Lemeshow statistic exceeding 0.05 across all five models, indicating an acceptable fit. Sensitivity analysis yielded similar results, further corroborating the reliability of the findings (Table S2). Further ROC curve analysis shows that the optimal cutoff values for CRP and platelets were determined to be 61.5 mg/L (Figure S1). The cutoff point for CRP was determined to be 61.95 mg/L. And a 10 mg/L increase in CRP concentration is correlated with a 7.2% heightened risk of CA development. The sensitivity and specificity at the cutoff point were 76.6% and 42.8% (Figure S1).

### 3.6. Follow-Up Echocardiography Data in Pediatric Patients With KD and CA

Follow-up echocardiographic information of 96 pediatric patients with KD and CA was recorded. Four patients (4.2%) developed thrombi in their coronary arteries. The thrombus in two patients (sAN and mAN) dissolved within 4 months; one thrombus in a patient remained unchanged in 6 years, while one patient was lost to follow-up.

**Figure 3:**
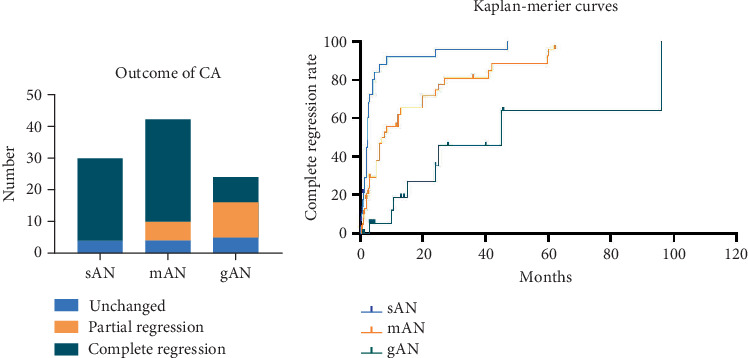
The regression of coronary aneurysm (CA) in three subgroups. (a) The number of CA regressions in patients with sAN, mAN, and gAN. (b) The Kaplan–Meier curves for the regression rate of CA by its severity. CA, coronary aneurysm; sAN, small aneurysms; mAN, medium aneurysms; gAN, giant aneurysms.

Thirteen patients (13.5%) exhibited no changes in CA, while partial regression of CA was observed in 17 patients (17.7%), and complete regression was noted in 66 patients (68.8%). In the sAN group, unchanged and complete regression of CA was noted in 4 (13.3%) and 26 (86.7%) patients, respectively, within 10.2 ± 11.5 months of follow-up time. For patients with mAN, unchanged, partial regression, and complete regression of CA were noted in 4 (9.5%), 6 (14.3%), and 32 (76.2%) patients, respectively, within 32.7 ± 27.9 months of follow-up time. For patients with gAN, unchanged, partial regression, and complete regression of CA were noted in 5 (20.8%), 11 (45.8%), and 8 (33.3%) patients, respectively, within 32.5 ± 22.8 months of follow-up time (Figure 3a).

### 3.7. Time-Dependent Changes of Regression Rates

For complete regression, in the sAN group, six children exhibited complete regression within 1 month, 13 within 3 months, five within 6 months, and one within 12 months. In the mAN group, 17 children achieved complete regression within 6 months, five within 12 months, four within 24 months, and six within a period ranging from 2 to 5 years. In the gAN group, one child achieved complete regression within 6 months, two within 12 months, two within 24 months, and three within a period ranging from 2 to 8 years (Figure 3b).

For partial regression, in the mAN group, partial regression of the coronary arteries was observed in three pediatric patients within 3 months. Additionally, one patient exhibited partial regression within 1 year, while two patients demonstrated partial regression over a span of 2 to 5 years. In the gAN group, four pediatric patients showed partial regression within 6 months. Additionally, four patients exhibited partial regression within 2 years, while three patients demonstrated partial regression over a span of 2 to 4 years (Table S3). Unchanged CA was observed in 13 patients, and most of the follow-up period was conducted with 1-month intervals (Table S3).

### 3.8. Regression in Affected Branches

**Figure 4:**
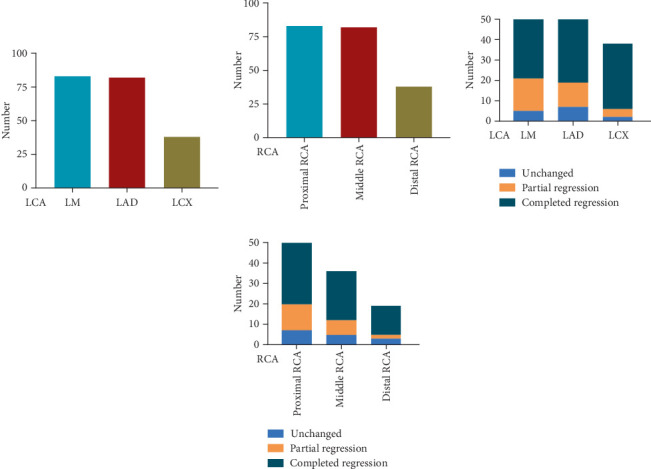
The regression of affected branches in the left coronary artery (LCA) and the right coronary artery (RCA). (a) Number of affected branches in LCA. (b) Number of affected branches in RCA. (c) The regression of affected branches in LCA. (d) The regression of affected branches in RCA. LCA, left coronary artery; RCA, right coronary artery; LM, left main coronary artery; LAD, left anterior descending artery.

Among the 336 coronary arteries affected in 96 patients, 203 (60.4%) branches in the LCA, mainly in the LM and LAD, and 133 (39.6%) branches in the RCA, mainly in the middle-proximal RCA, were affected (Figure 4a,b). During follow-up, the total complete regression rates of the LCA and RCA were 77.3% and 72.2%, respectively. Partial regression rates of the LCA and RCA were 15.8% and 16.5%, respectively (Figure 4c,d).

**Figure 5:**
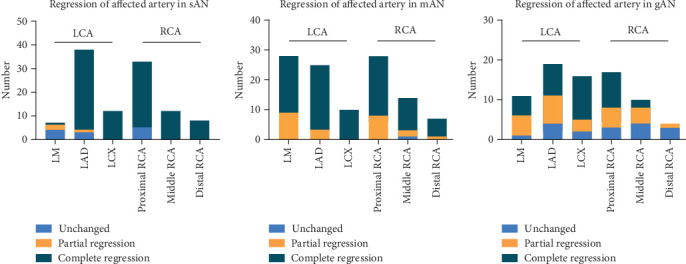
The regression of affected coronary arterial branches in the three subgroups. (a) Regression of the affected artery in sAN. (b) Regression of the affected artery in mAN. (c) Regression of the affected artery in gAN. sAN, small aneurysms; mAN, medium aneurysms; gAN, giant aneurysms; LCA, left coronary artery; RCA, right coronary artery; LM, left main coronary artery; LAD, left anterior descending artery; LCX, left circumflex.

In the regression of affected branches in the subgroups, the numbers of affected branches in the sAN, mAN, and gAN groups were 147, 112, and 77, respectively. Among patients with sAN, complete regression in LCA and RCA was noted in 84 (89.4%) and 48 (90.6%) branches, respectively (Figure 5a). Among patients with mAN, complete regression in LCA and RCA was noted in 51 (81.0%) and 37 (75.5%) branches, respectively (Figure 5b). In patients with gAN, complete regression in LCA and RCA was noted in 24 (52.2%) and 11 (31.4%) branches, respectively (Figure 5c).

## 4. Discussion

KD is an acute systemic vascular disease that predominantly affects medium and small vessels, particularly the coronary arteries. Approximately 80% of patients with KD are diagnosed before 5 years of age, with a peak incidence between 6 and 11 months [10]. However, KD in young patients, particularly those under the age of 1 year, is underdiagnosed and more likely to be incomplete KD [7, 11], leading to delayed treatment and a higher incidence of CA [12]. Moreover, patients aged <1 year have been shown to have CA [13]. Persistent CA may lead to adjacent stenosis or occlusion, resulting in ischemic heart disease [2]. Therefore, the early detection of risk factors for CA in patients with KD aged 1 year may be beneficial. In this context, the present study identified the risk factors for CA development in infants with KD and focused on CA regression. We identified male sex, duration of fever, illness days at initial IVIG treatment, and CRP level as risk factors for the development of CA in young patients with KD using multivariable analysis. Moreover, the regression and regression time of CA depended on the size of CA; however, giant CA in infants with KD showed a greater tendency to regress than that in older patients with KD.

Initial treatment with IVIG significantly reduces the incidence rate of CA; however, some patients still show CA development [14]. As such, it is meaningful to identify the potential risk factors for CA in patients with KD to identify more intensive treatments. In one systematic review and meta-analysis, sex, IVIG resistance, IVIG treatment beyond 10 days of symptom onset, and increased CRP level were all identified as risk factors for CA in patients with KD [15]. The presence of a greater number of the five typical symptoms of KD has been identified as a protective factor against CA [15]. In our previous study, male sex, age, fever duration, IVIG resistance, PLT count, ALB level, HB level, and ESR were all identified as predictors of medium- and giant-sized CA [16]. Moreover, age at disease onset <1 year is increasingly being considered a significant independent predictor of CA development [15, 17, 18], possibly due to the greater vulnerability of immature coronary arteries in infants to inflammation-related damage. However, the risk factors for CA development in young patients remain unclear. In our study, multivariate analysis revealed that male sex, fever duration, illness days at initial IVIG treatment, and CRP level were associated with the development of CA in infants with KD.

Epidemiological surveys have been conducted in different regions, and the reported male-to-female KD incidence ratios differ based on the country; however, a slight male predominance has been observed [19]. Male patients are at a high risk of IVIG resistance and CA development [5]. However, no explanation has been reported for this sex-specific bias, which may be owing to sex hormones, lifestyle, and behavior [20]. No obvious differences in estrogen levels have been observed between sexes in children [15]. Consequently, the estrogen level alone cannot be used to explain the incidence bias of CA in male patients with KD. In the present study, we also found a higher proportion of male patients with KD and CA, and male sex was identified as a risk factor for CA development.

Prolonged fever indicates sustained inflammation and not just severe inflammation, which seems to be associated with CA development [21]. In previous studies, duration of fever was significantly associated with CA development, with varying cutoff values reported, such as 8 [22], 10 [23], and 14 days [24], all showing associations with a high incidence of CA [25]. Consistent with many studies, prolonged fever duration (the sum of pre- and post-IVIG fever) was observed in the CA group and identified as a risk factor for CA development in patients with KD aged < 1 year. Therefore, treatment during the acute phase should be aimed at shortening fever duration by reducing systemic inflammation.

Immunomodulatory treatment in patients with IVIG reduces the overall risk of CA from 25% to 5% [14]. The incidence of CA regression was reported to be 55%–65% before immunoglobulin use, increasing to 75% with regular IVIG treatment [26, 27]. As such, the mainstay of initial treatment for KD includes a single high dose of IVIG. Additionally, the timing of IVIG initiation is also crucial. IVIG treatment within 10 days of KD onset has been shown to be effective at reducing the risk of CA formation from 25% to 3%–5% [8, 13]. Furthermore, some studies have indicated that later treatment within the 10-day window increases the risk of CA outcomes [28, 29]; however, one retrospective study found no significant difference in the development of adverse coronary outcomes regardless of the treatment day within 10 days of fever onset [30]. As such, IVIG treatment should be initiated as soon as possible after KD diagnosis. In our patients with KD with CA, the time to the first dose of IVIG was later than that in patients without CA. Meanwhile, more patients in the CA group initiated IVIG treatment >10 days after fever onset, possibly due to atypical symptoms and a delayed diagnosis. Furthermore, IVIG resistance was identified as an independent risk factor for CA in previous reports [31]. The present investigation revealed a markedly elevated incidence of IVIG resistance within the CA cohort compared to the non-CA cohort, although multivariate regression analysis failed to establish IVIG resistance as an independent risk factor for CA. The management of patients exhibiting resistance to IVIG remains challenging due to the lack of standardized therapeutic guidelines. In such cases, clinicians exercised their discretion to administer additional IVIG and/or steroids. In the present study, the majority of patients exhibiting IVIG resistance underwent a second dose of IVIG. Notably, a higher proportion of patients in the CA cohort received concurrent corticosteroid therapy compared to those in the non-CA cohort. The AHA guidelines recommend several therapeutic options, including a second IVIG infusion, MP, a prolonged tapering regimen of prednisolone or prednisone combined with IVIG, or the use of infliximab [8]. However, a systematic review has revealed no significant differences in the prevention of CA among patients treated with second IVIG infusion, infliximab, or intravenous pulse MP [32]. Furthermore, patients who fail to respond to these secondary interventions pose a distinct clinical challenge due to the absence of definitive guidelines for managing this subset of refractory KD cases [33]. To address this gap, a multicenter randomized controlled clinical trial is currently underway, aiming to evaluate the efficacy of various treatment strategies in KD patients unresponsive to a second dose of IVIG. The findings from this trial are expected to provide critical insights and inform subsequent therapeutic approaches for IVIG-resistant KD.

KD has no specific diagnostic laboratory parameters; however, NEU cell count, WBC count, HB, PLT count, CRP, transaminase, total bilirubin, NT-proBNP, ALB, sodium level, and ESR [16, 34–36] have been identified as risk factors for the development of CA in patients with KD. However, the associated risk factors of laboratory indicators can vary depending on the reports. Multivariate analysis revealed a significantly increased risk of CA in patients with elevated CRP levels. CRP is a nonspecific inflammatory biomarker that is elevated in response to inflammation. CRP levels generally correlate positively with the degree of inflammation and play a critical role in the acute phase of KD. CRP level has been identified as a predictor of IVIG resistance and serves as one of the criteria for confirming incomplete KD. Previous reports have indicated that a high CRP level (cutoff value of 105.5 mg/L) was associated with a higher risk of coronary arterial lesions (CALs) in patients with KD, which is consistent with the hypothesis that the inflammatory process is associated with CAL formation in patients with KD [37]. In the present study, the cutoff value of CRP was 61.95 mg/L, and the risk of CA escalates by 7.2% for every 10 mg/L increase in CRP level. As such, more attention should be paid to the clinical course of patients with KD with high CRP levels, particularly regarding the potential for CA formation.

KD-induced CAs are associated with the infiltration of the coronary artery wall by innate and adaptive immune cells, which leads to the subsequent release of proinflammatory cytokines and promotes vascular endothelial damage and the development of CA [38, 39]. In one multicenter prospective cohort study (RAISE) enrolling 125 patients with KD aged <1 year old (17.3% of the total cohort), 15 (12.0%) developed CA 1 month after disease onset, which was higher than the rate in patients aged >1 year (4.2%) [17]. In another retrospective cohort study including 2414 patients with KD, there were 3.6% patients under 1 year old and 2.6% patients older than 1 year old who developed CA, respectively [40]. In the present study, 18.8% of patients with KD aged <1 year developed CA, which was higher than both that patients aged <1 year and >1 year in previous reports. KD in infants, particularly those under 6 months of age, usually presents with atypical clinical manifestations [11]. Fever may be the sole clinical manifestation, accompanied by some other mild and transient symptoms. Atypical symptoms contribute to delays in both diagnosis and initiation of treatment. Furthermore, IVIG resistance has been identified as a critical factor for the development of CA [31]. In the present study, the prevalence of IVIG resistance was found to be 37.5% in the CA group and 20.4% in the non-CA group, both of which exceed the rates observed in older patients [7]. These findings underscore the heightened susceptibility of infant KD patients to developing CA compared to their older counterparts. As for the higher incidence of CA in our patients than in other reports with the same age group, this may be because not all patients with KD in our study were evaluated using the Kobayashi scoring system to predict IVIG sensitivity or resistance. In previous reports, patients who were evaluated as IVIG-resistant were initially treated with corticosteroids [41, 42] or infliximab [6] concurrent with IVIG. In the present study, we also found that patients in the non-CA group were more frequently initiated on treatment with IVIG combined with steroids compared to those in the CA group. Besides, the incidence of CA remains significant among infants under 6 months of age, even with prompt diagnosis and administration of IVIG. Specifically, nearly 20% of infants in this age group developed CA, in contrast to approximately 5% of infants 6 months or older [6]. And a study demonstrated that, in KD patients with an initial diagnosis of 2.5 ≤ *z* < 10, initial treatment with IVIG combined with corticosteroids or infliximab was more effective in mitigating the progression of CA size compared to IVIG monotherapy [43]. Therefore, among high-risk IVIG-resistant patients or the vulnerable population, adjunctive therapies may be beneficial for preventing CA development. In addition, variability in the inclusion criteria for CA across studies may contribute to differences in reported incidence rates. In the present study, CAs were defined as having a *z*‐score ≥ 2.5, whereas other studies employed more stringent thresholds, such as *z* ≥ 4 or *z* > 3 [26]. Furthermore, Takekoshi et al. reported a lower incidence of CA in infant patients [40], where CAs were characterized based on absolute internal lumen diameter. Evidence suggests that the use of *z*-score criteria results in a higher reported incidence of coronary abnormalities [26]. Additionally, CA was assessed 1 month following the onset of KD, and patients whose CA regressed within this 1-month period were considered transient dilation and subsequently excluded from the analysis [40]. Notably, regression data revealed that 9.4% of patients with CA experienced resolution within 1 month, particularly among those with sANs.

The CA usually occurs in the second week after the onset of KD, which can still increase after being afebrile to maximum dilation in the seventh week after the onset of KD due to the gradually vanished vasculitis [25]. Previous study reported that the median time of CA appearance and maximum diameter were 10 (4–34 days) and 35 (11–87 days) days, respectively [44]. The median time for initial dilation (*z* > 2), CA formation, and maximum dilation in our young patients was 10 (4–44 days), 13 (4–44 days), and 16 (4–432 days) days, respectively. This result indicated that the temporal interval from the initial formation to the peak development of CA in infants is markedly shorter compared to the whole KD cohort. In addition, more than 60% of the patients developed medium and giant CA, which agrees with the results of prior reports [43]. Furthermore, most CA cases are associated with bilateral coronary lesions, and the larger the CA, the higher the proportion of affected bilateral coronary arteries and branches. These data indicate a more serious inflammatory reaction, manifested as higher levels of inflammatory markers, leading to the rapid progression of CA in young patients. Coronary artery abnormalities range from dilation only to aneurysms of various numbers, sizes, and characteristics, with involvement occurring first in the proximal segments and then extending distally. Distal involvement without abnormalities in the proximal segments is rare. In the present study, we found that the LCA accounted for 60% of the affected coronary arteries, primarily including the LM and LAD, whereas the affected RCA was mainly concentrated in the proximal and middle RCA.

CA affects the prognosis of patients with KD. The regression of persistent CA relieves family anxiety and reduces the cost of healthcare. Factors associated with aneurysm regression include the age of diagnosis <1 year, small size (<5 mm ID), fusiform morphology, and distal coronary segment involvement [45, 46]. Data indicate that patients with small CAs of less than 6 mm or *z* < 5 can be regressed, and patients with median CA less than 8 mm or 5 ≤ *z* < 10 have about 30% chance to regress, while patients with giant CA more than 8 mm or *z* > 10 are less than 10% chance to experience regression [45, 46]. In addition, diagnosis under the age of 1 year was associated with a high regression rate, and even in larger CA, the regression rate was more than half [27]. Regarding the regression of affected branches, in the largest Japanese study with the longest follow-up of CA, including 1006 patients with KD aged <19 years, the overall regression rates during the study period were 68% in the RCA group and 70.9% in the LAD group. Moreover, the regression rates were 95.5% for small, 83.2% for medium, and 36.3% for larger RCAs and 95.3% for small, 80.1% for medium, and 28.8% for larger LADs [27]. In the subgroup of patients aged <1 year, the regression rates were 93.3%, 88.4%, and 53.3% for small, medium, and large CAs in the RCA and 100%, 88.6%, and 61.9% for small, medium, and large CAs in the LAD, respectively [27]. In the present study, 86.7%, 76.2%, and 33.3% of the patients with sAN, mAN, and gAN, respectively, showed complete regression. The complete regression rates were 90.6%, 75.5%, and 35.5% for sAN, mAN, and gAN in the RCA and 89.4%, 81.0%, and 52.2% for sAN, mAN, and gAN in LCA, respectively. These data indicate that the regression rates of CA in patients aged <1 year were higher than previously reported in patients with KD but lower than previously reported in patients aged <1 year. First, CA regression may result from intimal thickening predominantly caused by the proliferation of smooth muscle cells [47], whereas the ability of smooth muscle cells to proliferate is lost with age [48]. Consequently, the higher regression rate in infants with KD may be partially associated with the altered ability of muscle cells. Second, the follow-up period for this study, particularly concerning giant CAs, was relatively short. A higher regression rate in younger patients with KD was reported in one 10-year follow-up study [27]. Even though the time to regression was reported to be within 2 years, the regression of large CA was observed even after >2 years [26, 27]. And regression median time was reported to be 136 days with a range of 41–386 days. Notably, 78% of cases regressed within 200 days [44]. In the present study, 58.3% of patients experienced complete regression within 2 years, while 10.4% patients recovered beyond 2 years. In addition, 14.3% and 45.8% of patients with mAN and gAN, respectively, showed a tendency for regression (partial regression) to low classifications. Therefore, the median follow-up of 32 months primarily reflects short- to mild-term outcomes, and extended follow-up remains necessary to comprehensively assess long-term prognoses of CAs, particularly gANs. Third, in our institution, echocardiographic evaluations and follow-up assessments were performed more frequently than the previously established protocol, particularly during the acute phase [6]. This intensified frequency of imaging and monitoring enabled a more rapid and comprehensive detection of alterations in CAs and the progression of associated complications, thereby facilitating timely intervention and better prognosis. Furthermore, the regression of large aneurysms does not guarantee that no cardiovascular events will occur; as such, the follow-up time in infant patients, particularly patients with giant CA, should be long because of the constantly changing physiology. Based on the present study, in clinical practice, parents should be informed that small and medium CAs generally have a benign outcome, which might reduce their anxiety regarding prognoses; giant CAs in infant patients with KD are more likely to regress than those in older patients.

This study has some limitations that necessitate caution in its interpretation. First, treatments for IVIG-resistant patients were not standardized, which may have affected the development and prognosis of CA. Further clinical investigations are warranted to establish uniform treatment protocols. Second, the follow-up period was relatively short for some patients, particularly patients with gANs. Future studies with substantially longer follow-up durations (e.g., 5-year or 10-year) are essential to definitively establish the long-term prognosis of gANs. However, this observation at this intermediate point remains a clinically significant and encouraging finding, which primarily reflects the short-to-mid-term efficacy and safety profile of the current treatment and regression of CAs. Third, the timing of coronary angiography was not standardized, which may have affected the precise timing of confirmation of CA regression. Last, despite adjustment for potential confounders, residual confounding may persist due to the retrospective observational nature of this study.

## 5. Conclusions

In this study, we found that the incidence of CA is higher in infants with KD than in other age groups. Therefore, the early diagnosis, identification of risk factors for CA, and improvement of acute-phase treatment in this population would help reduce the risk of CA occurrence, which would reduce families' anxiety and the burden on society. The present study demonstrated a correlation between male sex, fever duration, illness days for initial IVIG, CRP level, and the occurrence of CA. In addition, the follow-up data indicated that small and medium CAs have a greater chance of complete regression, and giant CAs in infant patients with KD have a greater tendency to regress than those in older patients with KD; however, a long follow-up time is required for validation of these findings.

## Figures and Tables

**Table 1:** Demographic features of patients with KD in the non-CA and CA groups.

**Characteristic**	**Non-CA group (** **n** = 285**)**	**CA group (** **n** = 96**)**	**p** ** value**
Referral patients	22	35	NA
Diagnosed with CA at the time of referral	—	19	NA
Age (months)	6.7 ± 2.9	6.7 ± 3.0	0.79
Male (*n*, %)	187 (65.6)	69 (71.9)	0.26
Weight (kg)	8.2 ± 1.5	7.9 ± 1.3	0.06
Duration of fever (days)	7.1 ± 2.7	9.9 ± 3.4	<0.01
Illness days of initial IVIG (days)	6.2 ± 2.0	8.8 ± 4.1	<0.01
Initial IVIG <10 days of illness (*n*, %)	266 (93.3)	59 (61.5)	<0.01
Type of KD			
Incomplete KD (*n*, %)	70 (24.6)	37 (38.5)	0.01
IVIG-resistant KD (*n*, %)	58 (20.4)	36 (37.5)	<0.01
Initial therapy combined with GCS (*n*, %)	27 (9.5)	5 (5.2)	0.19
Frequency of the classical signs			
With fever (*n*, %)	285 (100.0)	96 (100.0)	NA
Bilateral conjunctival congestion (*n*, %)	265 (93.0)	81 (84.4)	0.01
Changes in the lips and oral mucosa (*n*, %)	259 (90.9)	79 (82.3)	0.02
Polymorphic rash (*n*, %)	254 (89.1)	75 (78.1)	0.01
Asymmetric lymph node enlargement (*n*, %)	164 (57.5)	54 (56.3)	0.30
Swelling and peeling of the fingertips and distal toes (*n*, %)	202 (70.9)	61 (63.5)	0.11
Peripheral artery dilation or aneurysms (*n*, %)	0 (0.0%)	9 (9.4%)	NA

*Note:* Values are presented as a number (%) or mean ± standard deviation. *p* value < 0.05, statistically significant.

Abbreviations: CA, coronary aneurysm; IVIG, intravenous immunoglobulin; KD, Kawasaki disease; NA, not applicable.

**Table 2:** Treatments in patients diagnosed with Kawasaki disease in the non-CA and CA groups.

**Combination therapy**	**Non-CA group (** **n** = 285**)**	**CA group (** **n** = 96**)**
Initial IVIG treatment		
First dose of IVIG without steroid (*n*, %)	258 (90.5)	91 (94.8)
First dose of IVIG concomitant with steroid (*n*, %)	27 (9.5)	5 (5.2)
IVIG-resistant patients	*n* = 58	*n* = 36
Second dose of IVIG (*n*, %)	48 (82.8)	31 (86.1)
Second dose of IVIG and concomitant with steroid (*n*, %)	23 (47.9)	22 (71.0)
Second dose of IVIG only (*n*, %)	25 (52.1)	9 (29.0)
Treated with steroid only (*n*, %)	10 (17.2)	5 (13.9)
Third dose of IVIG (*n*, %)	5 (8.6)	10 (27.8)
Antithrombosis treatment		
Patient without an aneurysm		
Single antiplatelet (*n*, %)	125 (43.9)	—
Dual antiplatelet (*n*, %)	160 (56.1)	—
Patients with sAN (*n* = 32)		
Single antiplatelet (*n*, %)	—	11 (34.4)
Dual antiplatelet (*n*, %)	—	21 (65.6)
Patients with mAN (*n* = 40)		
Single antiplatelet (*n*, %)	—	4 (10.0)
Dual antiplatelet (*n*, %)	—	32 (80.0)
Warfarin and dual antiplatelet (*n*, %)	—	4 (10.0)
Patients with gAN (*n* = 24)		
Warfarin and single antiplatelet (*n*, %)	—	10 (41.7)
Dual antiplatelet (*n*, %)	—	8 (33.3)
Warfarin and dual antiplatelet (*n*, %)	—	6 (25.0)

*Note:* Single antiplatelet treatment includes aspirin, dipyridamole, or clopidogrel. Dual antiplatelet treatment includes aspirin and dipyridamole.

Abbreviations: CA, coronary aneurysm; gAN, giant aneurysm; IVIG, intravenous immunoglobulins; KD, Kawasaki disease; mAN, medium aneurysm; sAN, small aneurysm.

**Table 3:** Laboratory features of patients with Kawasaki disease in the non-CA and CA groups.

**Laboratory results**	**Non-CA group**	**CA group**	**p** ** value**
WBC (×10^9^/L)	17.3 ± 5.9	18.7 ± 6.4	0.06
NEU (×10^9^/L)	10.0 ± 4.6	15.7 ± 48.0	0.26
LYM (×10^9^/L)	5.3 ± 2.4	6.0 ± 3.0	0.08
HB (g/L)	101.3 ± 11.8	98.5 ± 13.2	0.06
PLT (×10^9^/L)	444.7 ± 195.0	548.0 ± 268.4	<0.01
CRP (mg/L)	79.6 ± 44.6	99.2 ± 58.6	<0.01
ALB (g/L)	36.2 ± 4.4	34.3 ± 5.6	<0.01
TP (g/L)	60.5 ± 6.1	62.0 ± 8.4	0.09
Na^+^ (mmol/L)	134.9 ± 2.2	135.0 ± 2.2	0.77
LDH (IU/L)	331.0 ± 93.2	319.1 ± 184.9	0.57
ESR (mm/H)	49.0 ± 23.3	59.6 ± 26.5	<0.01
Ferritin (ng/mL)	239.5 ± 178.7	226.7 ± 170.9	0.59
PCT (ng/mL)	1.70 ± 5.60	0.9 ± 1.6	0.17
ALT (IU/L)	60.9 ± 104.9	68.9 ± 132.4	0.55
AST (IU/L)	63.8 ± 118.7	73.8 ± 100.5	0.47

*Note:* Values are presented as mean ± standard deviation. *p* value < 0.05 is statistically significant.

Abbreviations: ALB, albumin; ALT, alanine aminotransferase; AST, aspartate aminotransferase; CA, coronary aneurysm; CRP, C-reactive protein; ESR, erythrocyte sedimentation rate; HB, hemoglobin; LDH, lactic dehydrogenase; LYM, lymphocyte; NEU, neutrophil; PCT, procalcitonin; PLT, platelets; TP, total protein; WBC, white blood cell count.

**Table 4:** Echocardiographic evaluation of patients diagnosed with Kawasaki disease with coronary aneurysm.

**CA type**	**All CAs (** **n** = 96**)**	**sAN (** **n** = 30**, 31.2%)**	**mAN (** **n** = 42**, 43.8%)**	**gAN (** **n** = 24**, 25.0%)**
Start dilation time (median, range), day	10, 4–44	9, 4–26	10, 4–44	13, 4–26
Formation time (median, range), day	13, 4–44	9, 4–26	10, 5–44	14.5, 4–30
Maximum dilation (median, range), day	16, 4–432	9.5, 4–30	14, 5–47	25, 4–432

Abbreviations: CA, coronary aneurysm; gAN, giant aneurysm; LCA, left coronary artery; mAN, medium aneurysm; RCA, right coronary artery; sAN, small aneurysm.

**Table 5:** Multivariate regression analysis for coronary aneurysm among patients with Kawasaki disease.

	**Multivariate analysis**		
**Covariates**	**OR**	**95% CI**	**p** ** value**
**Sex: Male**	**1.982**	**1.037–3.788**	**0.04**
Weight	0.807	0.605–1.076	0.14
Age	1.049	0.911–1.207	0.51
**Duration of fever**	**1.143**	**1.046–1.286**	**0.03**
**Illness days of initial IVIG**	**1.319**	**1.157–1.505**	**<0.01**
Complete KD	0.820	0.431–1.560	0.55
IVIG resistance	1.605	0.691–3.727	0.27
White blood cell count (×10^9^ L)	0.911	0.742–1.119	0.35
Neutrophil count (×10^9^ L)	1.084	0.867–1.355	0.44
Lymphocyte count (×10^9^ L)	1.139	0.888–1.461	0.29
Platelet count (×10^9^ L)	1.000	0.998–1.001	0.57
**C-reactive protein (mg/L)**	**1.007**	**1.001–1.013**	**0.02**
Hypoproteinemia (<30 g/L)	1.170	0.594–2.304	0.65
Total protein (g/L)	1.028	0.983–1.075	0.22
Low hemoglobin (<95 g/L)	1.170	0.594–2.304	0.65
Abnormal procalcitonin (>0.5 ng/mL)	0.728	0.386–1.372	0.33
AST/ALT ratio	1.047	0.733–1.496	0.80

*Note:* Boldface text denotes significance. ORs were adjusted for weight, age, complete KD, IVIG resistance, white blood cell count, neutrophil count, lymphocyte count, platelet count, hypoproteinemia, total protein, low hemoglobin, abnormal procalcitonin, and AST/ALT ratio. *p* value < 0.05 indicates statistical significance.

Abbreviations: AST/ALT, aspartate aminotransferase/alanine transaminase ratio; IVIG, intravenous immunoglobulin; KD, Kawasaki disease; OR, odds ratio.

## Data Availability

The data that support the findings of this study are available on request from the corresponding author. The data are not publicly available due to privacy or ethical restrictions.

## Nomenclature

AHA: American Heart Association
ALB: albumin
ALT: alanine aminotransferase
AST: aspartate aminotransferase
BNP: brain natriuretic peptide
CA: coronary aneurysm
CALs: coronary arterial lesions
CRP: C-reactive protein
ESR: erythrocyte sedimentation rate
FDP: fibrinogen degradation products
FER: ferritin
gAN: giant aneurysm
HB: hemoglobin
IVIG: intravenous immunoglobulin
KD: Kawasaki disease
LAD: left anterior descending artery
LCX: left circumflex
LDH: lactic dehydrogenase
LM: left main coronary artery
LYM: lymphocyte
mAN: medium aneurysm
MP: methylprednisolone
NEU: neutrophil
OR: odds ratio
PCT: procalcitonin
PLT: platelet
RCA: right coronary artery
sAN: small aneurysm
SD: standard deviations
TP: total protein
WBC: white blood cells

## Supporting Information

Supporting Information Additional supporting information can be found online in the Supporting Information section. Table S1. Univariate regression analysis for coronary aneurysm. Table S2. Sensitive analysis of multivariate regression analysis for coronary aneurysm. Table S3. Regression of coronary aneurysm during follow-up. Figure S1. The receiver operating curve of C-reactive protein (CRP). The cutoff point for CRP was determined to be 61.95 mg/L. The sensitivity and specificity at the cutoff point were 76.6% and 42.8%.
