# Supplementary material for: Risk Factors and Regression of Coronary Aneurysms in Infants With Kawasaki Disease
Source: Cardiovasc Ther. 2025 Nov 11;2025:9988778. doi: 10.1155/cdr/9988778 (PMC12626698; doi:10.1155/cdr/9988778)

Supplementary Table 1. Univariate regression analysis for coronary aneurysm.

Supplementary Table 2. Sensitive analysis of multivariate regression analysis for coronary aneurysm.

Supplementary Table 3. Regression of coronary aneurysm during follow-up.

Supplementary Figure 1. The receiver operating curve of C-reaction protein (CRP). The cutoff point for CRP was determined to be 61.95 mg/L. The sensitivity and specificity at the cutoff point were 76.6% and 42.8%.

**Supplementary Table 1. Univariate regression analysis for coronary aneurysm**

|  | Univariate analysis | | |
| --- | --- | --- | --- |
| Covariates | OR | 95% CI | P-value |
| Sex: male | 1.339 | 0.806–2.225 | 0.259 |
| Weight | 0.879 | 0.749–1.031 | 0.113 |
| Age | 0.990 | 0.915–1.07 | 0.990 |
| Duration of fever | 1.327 | 1.220–1.442 | <0.001 |
| Illness days of initial IVIG | 1.364 | 1.241–1.498 | <0.001 |
| Initial IVIG >10 days of illness | 8.207 | 4.719–16.336 | <0.001 |
| Complete KD | 0.519 | 0.31–0.849 | 0.009 |
| IVIG resistance | 2.348 | 1.419–3.887 | <0.001 |
| WBC (×10^9^ L) | 1..037 | 0.999-1.076 | 0.058 |
| NEU (×10^9^ L) | 1.031 | 0.982–1.083 | 0.215 |
| LYM (×10^9^ L) | 1.095 | 1.001-1.199 | 0.048 |
| PLT (×10^9^ L) | 1.002 | 1.001–1.003 | <0.001 |
| CRP (mg/L) | 1.008 | 1.003–1.012 | 0.001 |
| Hypoproteinemia (<30 g/L) | 2.804 | 1.497–5.255 | <0.001 |
| TP | 1.038 | 1.000-1.078 | 0.048 |
| Hyponatremia (<135 mmol/L) | 0.799 | 0.489–1.301 | 0.369 |
| Lower HB (<95 g/L） | 2.528 | 1.551–4.122 | <0.001 |
| ALT (U/L) | 1.001 | 0.999-1.003 | 0.553 |
| AST (U/L) | 1.001 | 0.999-1.003 | 0.467 |
| AST/ALT ratio | 1.322 | 1.024-1.706 | 0.032 |
| PCT (>0.5 ng/ml) | 0.610 | 0.367–1.013 | 0.056 |
| Ferritin (ng/ml) | 1.000 | 0.998-1.001 | 0.588 |
| LDH | 0.999 | 0.997-1.002 | 0.455 |
| ESR higher (mm/h) * | 3.510 | 0.446-27.597 | 0.233 |

IVIG, intravenous immunoglobulin; KD, Kawasaki disease; WBC, white blood cell; NEU, neutrophil; LYM, lymphocyte; PLT, platelet count, CRP, C-reactive protein; TP, total protein; HB, hemoglobin; ALT, alanine aminotransferase; AST, aspartate aminotransferase; PCT, procalcitonin; LDH, lactic dehydrogenase; ESR, erythrocyte sedimentation rate.

*ESR, >15 mm/h for male and >20 mm/h for female

*P*-value <0.05 indicates statistical significance.

**Supplementary Table 2. Sensitive analysis of multivariate regression analysis for coronary aneurysm**

|  | Multivariate analysis | | |
| --- | --- | --- | --- |
| Covariates | OR | 95% CI | P-value |
| **Sex: male** | **2.384** | **1.162-4.893** | **0.018** |
| Weight | 0.801 | 0.528-1.102 | 0.172 |
| Age | 1.030 | 0.882-1.201 | 0.711 |
| **Duration of fever** | **1.258** | **1.079-1.467** | **0.003** |
| **Illness days of initial IVIG** | **1.199** | **1.031-1.393** | **0.018** |
| Complete KD | 0.871 | 0.434-1.749 | 0.698 |
| IVIG resistance | 1.060 | 0.398-2.821 | 0.907 |
| WBC (×10^9^ L) | 0.793 | 0.621-1.628 | 0.063 |
| NEU (×10^9^ L) | 1.258 | 0.968-1.725 | 0.082 |
| LYM (×10^9^ L) | 1.292 | 0.968-1.725 | 0.082 |
| PLT (×10^9^ L) | 1.000 | 0.998-1.001 | 0.662 |
| CRP (mg/L) | 1.004 | 0.997-1.011 | 0.277 |
| Hypoproteinemia (<30 g/L) | 1.035 | 0.986-1.087 | 0.167 |
| TP | 1.035 | 0.986-1.087 | 0.167 |
| HGB (<95 g/L） | 1.441 | 0.689-3.011 | 0.332 |
| PCT (>0.5 ng/ml) | 0.704 | 0.339-1.462 | 0.346 |
| AST/ALT | 1.263 | 0.883-1.807 | 0.201 |

IVIG, intravenous immunoglobulin; KD, Kawasaki disease; WBC, white blood cell; NEU, neutrophil; LYM, lymphocyte; PLT, platelet count, CRP, C-reactive protein; TP, total protein; HB, hemoglobin; ALT, alanine aminotransferase; AST, aspartate aminotransferase; PCT, procalcitonin; LDH, lactic dehydrogenase.

*P*-value <0.05 indicates statistical significance.

Missing data were deleted directly in this analysis, there were Nagelkerke R square is 0.355, Hosmer and Lemeshow Test P value is 0.352. There were 33 patients not included in the model.

**Supplementary Table 3. Regression of coronary aneurysm during follow-up**

|  |  | **Outcome of coronary aneurysm** | | | | | |
| --- | --- | --- | --- | --- | --- | --- | --- |
|  |  | **Unchanged** | | **Partial regression** | | **Complete regression** | |
| **CA type** | **Follow-up time (month)** | **Frequency (n, %)** | **Follow-up time* (month)** | **Frequency (n, %)** | **Regression time* (month)** | **Frequency (n, %)** | **Regression time* (month)** |
| **sAN (n=30)** | 10.2±11.5 | 4 (13.3%) | 0.6±0.3 | 0 (0.0%) | - | 26 (86.7%) | 3.9±9.2 |
| **mAN (n=42)** | 32.7±27.9 | 4 (9.5%) | 17.6±21.3 | 6 (14.3%) | 4.5±2.8 | 32 (76.2%) | 12.8±17.2 |
| **gAN (n=24)** | 32.5±22.8 | 5 (20.8%) | 26.1±25.7 | 11 (45.8%) | 15.7±13.4 | 8 (33.3%) | 20.7±11.2 |

Values are presented as n (%) or mean ± standard deviation

CA, coronary aneurysm; gAN, giant aneurysm; mAN, medium aneurysm; sAN, small aneurysm

^*^Time was defined as the first time that the CA regressed to a minimum size and was maintained

**Supplementary Figure 1**
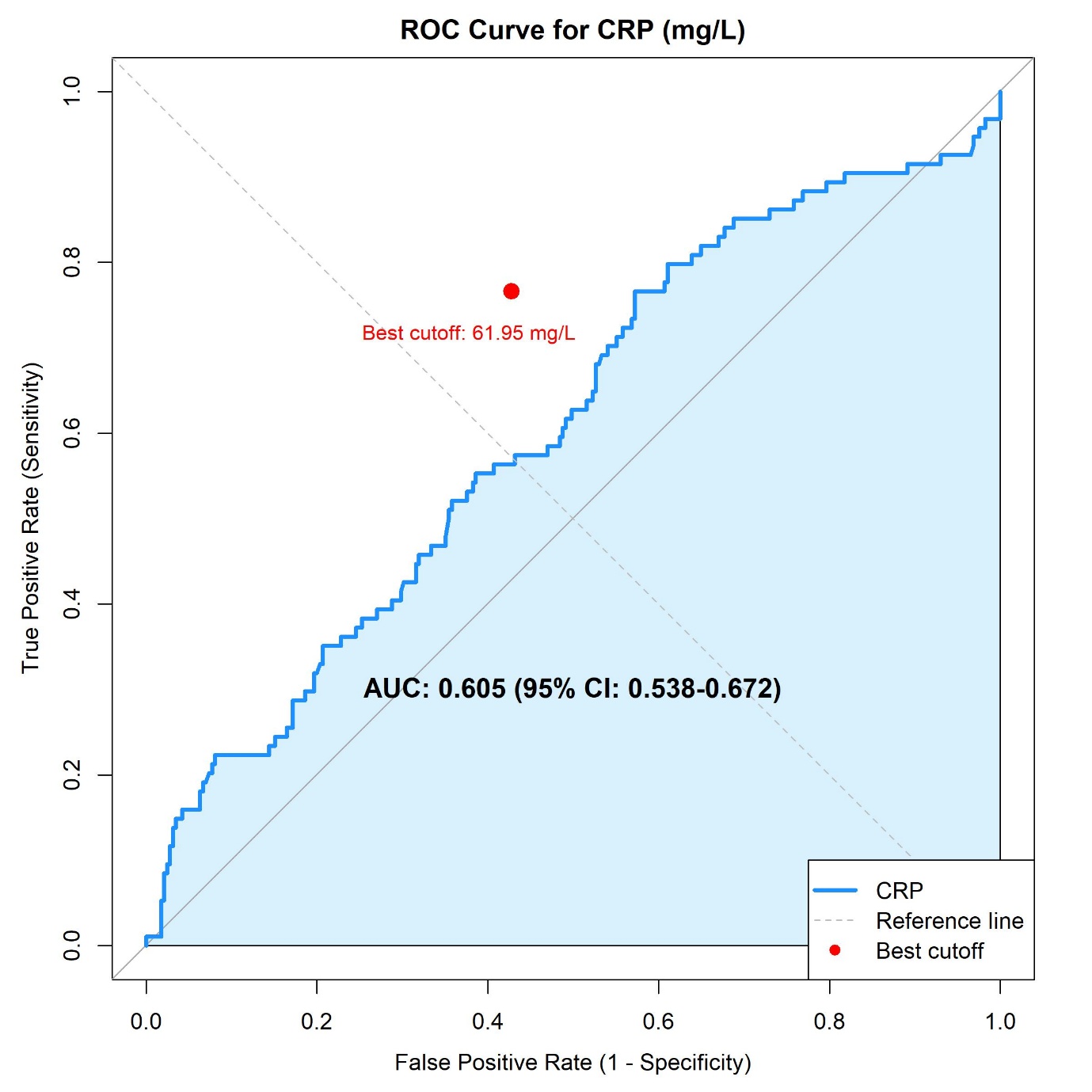

Supplement: Supporting Information — Additional supporting information can be found online in the Supporting Information section. Table S1. Univariate regression analysis for coronary aneurysm. Table S2. Sensitive analysis of multivariate regression analysis for coronary aneurysm. Table S3. Regression of coronary aneurysm during follow-up. Figure S1. The receiver operating curve of C-reactive protein (CRP). The cutoff point for CRP was determined to be 61.95 mg/L. The sensitivity and specificity at the cutoff point were 76.6% and 42.8%. [file 9988778.f1.docx]
